# Competition for Antigen between Th1 and Th2 Responses Determines the Timing of the Immune Response Switch during *Mycobaterium avium* Subspecies *paratuberulosis* Infection in Ruminants

**DOI:** 10.1371/journal.pcbi.1003414

**Published:** 2014-01-09

**Authors:** Gesham Magombedze, Shigetoshi Eda, Vitaly V. Ganusov

**Affiliations:** 1 National Institute for Mathematical and Biological Synthesis, University of Tennessee, Knoxville, Tennesse, United States of America; 2 Department of Forestry, Wildlife, and Fisheries, University of Tennessee, Knoxville, Tennesse, United States of America; 3 Department of Microbiology, University of Tennessee, Knoxville, Tennesse, United States of America; 4 Department of Mathematics, University of Tennessee, Knoxville, Tennesse, United States of America; Emory University, United States of America

## Abstract

Johne's disease (JD), a persistent and slow progressing infection of ruminants such as cows and sheep, is caused by slow replicating bacilli *Mycobacterium avium* subspecies *paratuberculosis* (MAP) infecting macrophages in the gut. Infected animals initially mount a cell-mediated CD4 T cell response against MAP which is characterized by the production of interferon 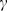 (Th1 response). Over time, Th1 response diminishes in most animals and antibody response to MAP antigens becomes dominant (Th2 response). The switch from Th1 to Th2 response occurs concomitantly with disease progression and shedding of the bacteria in feces. Mechanisms controlling this Th1/Th2 switch remain poorly understood. Because Th1 and Th2 responses are known to cross-inhibit each other, it is unclear why initially strong Th1 response is lost over time. Using a novel mathematical model of the immune response to MAP infection we show that the ability of extracellular bacteria to persist outside of macrophages naturally leads to switch of the cellular response to antibody production. Several additional mechanisms may also contribute to the timing of the Th1/Th2 switch including the rate of proliferation of Th1/Th2 responses at the site of infection, efficiency at which immune responses cross-inhibit each other, and the rate at which Th1 response becomes exhausted over time. Our basic model reasonably well explains four different kinetic patterns of the Th1/Th2 responses in MAP-infected sheep by variability in the initial bacterial dose and the efficiency of the MAP-specific T cell responses. Taken together, our novel mathematical model identifies factors of bacterial and host origin that drive kinetics of the immune response to MAP and provides the basis for testing the impact of vaccination or early treatment on the duration of infection.

## Author Summary

*Mycobacterium avium* subsp. *paratuberculosis* (MAP) is the causative agent of Johne's disease, a chronic enteric disease of ruminants such as sheep and cows. Due to early culling and reduction in milk production of affected animals, MAP inflicts high economic cost to diary farms. MAP infection has a long incubation period of several years, and during the asymptomatic stage a strong cellular (T helper 1) immune response is thought to control MAP replication. Over time, Th1 response is lost and ineffective antibody response driven by Th2 cells becomes predominant. We develop the first mathematical model of helper T cell response to MAP infection to understand impact of various mechanisms on the dynamics of the switch from Th1 to Th2 response. Our results suggest that in contrast to the generally held belief, Th1/Th2 switch may be driven by the accumulation of long-lived extracellular bacteria, and therefore, may be the consequence of the disease progression of MAP-infected animals and not its cause. Our model highlights limitations of our current understanding of regulation of helper T cell responses during MAP infection and identifies areas for future experimental research.

## Introduction

*Mycobacterium avim* subsp. *paratuberculosis* (MAP) infects intestine of ruminants (e.g., cattle and sheep) and causes a chronic inflammatory disease called Johne's disease (JD) [1], [2]. Due to reduction of milk production and early culling of diseased animals, JD causes a significant economic loss to animal industries [3], [4]. MAP has also been suspected as a causative agent of Crohn's disease, an inflammatory bowel disease in human [5].

Infection of animals occurs mainly through ingestion of materials contaminated with MAP-containing feces [6]. After the ingestion MAP bacilli reach intestine of the animal, are taken up by M cells and enterocytes, and are engulfed by submucosal macrophages [7]–[10]. MAP survives in resting tissue macrophages by inhibiting phagosome maturation [11]–[15]. At late stages of JD, MAP-infected animals shed bacilli in their feces thereby completing the infection cycle.

**Figure 1:**
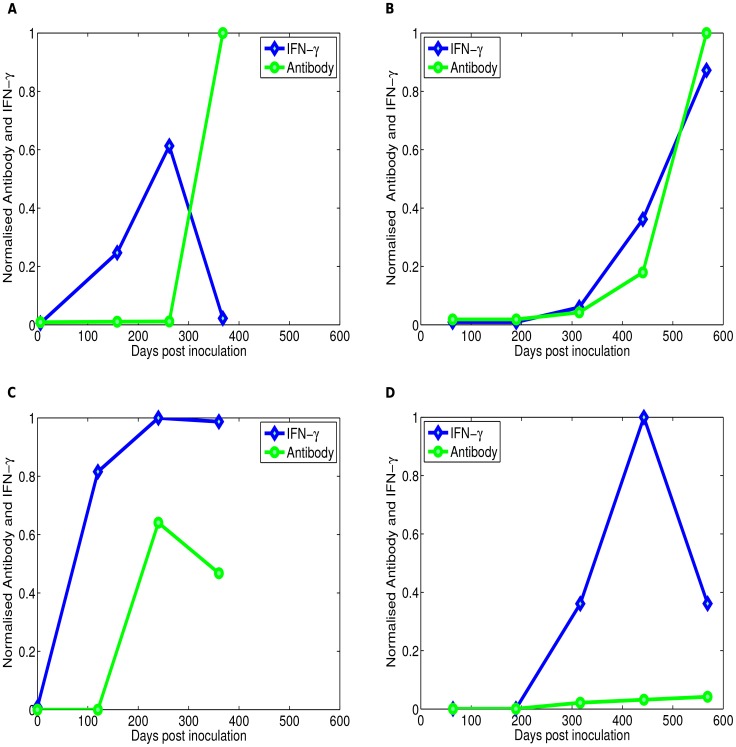
Experimental data on the kinetics of immune response during experimental MAP infection. The kinetics of bacteria-specific cellular (Th1) and antibody (Th2) responses during experimental infection of sheep with MAP [20]. The immune response data was digitised from [20]. Th1 response was measured as the concentration of IFN-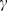 produced after stimulation of peripheral blood mononuclear cells with MAP antigens. Th2 response was measured as the titer of MAP-specific antibodies in the blood. In both cases, immune responses were normalised to a positive control and thus range from 0 to 1. Four different patterns of the Th1/Th2 responses have been observed including a “classical” switch (panel **A**, observed in 39% of infected sheep), a combined response (panels **B**& **C**, observed in 50% of animals), and only Th1 response (panel **D**, observed in 11% of infected animals).

MAP infection follows a lengthy latent and sub-clinical period in which the infection is difficult to diagnose [1], [2]. Current research efforts focus on developing tools that aide in early detection of the infection before the infected animals start shedding MAP into the environment [16]. Vaccines are available for ovine and bovine JD [17]. Although the vaccines reduce or delay clinical symptoms and shedding of MAP into feces, they do not prevent new infections [17]. The lack of progress in vaccine development is in part due to poor understanding of the nature of the protective immune response against MAP infection [18]. In this respect, experimental infections of animals with MAP have been carried out in several studies examining the kinetics of MAP-specific immune responses [18]–[20]. These studies demonstrated that animals with paucibacillary lesions (at early stages of the infection) are likely to express a cell mediated (Th1-type) immune response measured by the expression of IFN-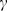 [21]. This response is likely to be protective against intracellular pathogens since IFN-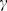 can induce intracellular killing of MAP by macrophages [22], [23], and infected animals with a dominant Th1 response have very few lesions [20]. At later stages of the infection animals with multi-bacillary lesions express predominantly a Th2-type immune response that is measured by the presence of MAP-specific IgG1 antibodies, production of which is driven by IL-4 or IL-10 producing CD4 T cells [18]–[20]. Although high levels of MAP-specific antibodies are detected in animals in late stages of the disease, these antibodies do not appear to be protective and may even be detrimental by increasing uptake of extracellular bacteria by macrophages [24]. Thus, experimental infections of sheep with MAP suggest a switch from dominance of the MAP-specific Th1 immune response in the early stages of the disease to a predominantly Th2 response at later stages of the disease (Figure 1A).

More detailed analysis of the kinetics of MAP-specific Th1 (IFN-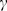) and Th2 (antibody) responses in experimentally infected sheep revealed that the majority of animals do not display the “classical” Th1/Th2 switch (Figure 1B–D). Around 50% of infected animals have combined Th1/Th2 responses (Figure 1B&C) while the minority of animals (11%) show only Th1 response (Figure 1D). Reasons for such different patterns of the kinetics of Th1/Th2 responses are not well understood.

Th1 and Th2 subsets of helper CD4 T cell responses are defined by a set of cytokines they secrete and transcription factors that drive the development of each subset. Both Th1 and Th2 effectors differentiate from naïve CD4 T cells depending on the type of cytokines in the environment and the stimulating antigen [25], [26]. Interleukin 12 (IL-12), IFN-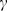, and strong antigenic stimulation upregulate expression of a transcription factor T-bet in naïve CD4 T cells that in turn drives differentiation of T cells into Th1 effectors. IL-4 and weak antigenic stimulation upregulate expression of a transcription factor GATA-3 that in turn drives differentiation of naïve T cells into Th2 effectors. Differentiated effector T cells themselves also start producing cytokines. Th1 effectors produce proinflammatory cytokines such as tumor necrosis factor-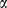 (TNF-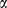) and IFN-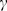. These cytokines activate macrophages to kill intracellular bacteria [13]. Th2 effectors produce a different group of cytokines such as IL-4, IL-5, IL-6, IL-10, and IL-13. Th2 effectors and these cytokines direct B cells to produce MAP-specific antibodies [26]. There is a competition between Th1 and Th2 responses as observed in *in vitro* experiments [27]–[29]. Cytokines produced by Th1 cells inhibit differentiation of naïve CD4 T cells into Th2 cells and *vice versa* [30].

Mathematical modelling has influenced the current understanding of Th1/Th2 cell differentiation [31]–[36]. These models can be divided into three categories (i) models that describe different T cell phenotypes induced by transcription factors that govern the molecular mechanism for lineage selection and maintenance [31]–[33], [37]–[39], (ii) differentiation of naïve T cells into a mixed population of Th1 and Th2 effectors in response to cytokines induced by antigen-presenting cells and each T cell subset [34]–[36], [40], [41], and (iii) regulatory network reconstruction with a repertoire of molecular and cellular factors that control Th cell differentiation [42]–[45]. As far as we know, only limited modelling work has been done on dynamics of Th1/Th2 responses to specific pathogens, such as viruses (e.g., human immunodeficiency virus) and mycobacterial pathogens (e.g., *Mycobacterium tuberculosis*). Most of mathematical models did not specify pathogen to simulate differentiation of naïve CD4 T cells into different Th cell subsets [31]–[36], [38], [41], [45].

The switch from a Th1 to a Th2 immune response in MAP-infected animals often occurs together with signs of clinical disease [20], [21]. Mechanisms underlying this switch are still poorly understood, in particular it is unclear 1) which factors contribute to the timing of the switch, 2) whether the timing of the switch can be regulated, and 3) whether the switch is the driver of infection to the clinical stage or it is just a consequence of the progression to clinical disease. To address these questions, we developed a mathematical model of the immune response to MAP infection. We use this model to understand and identify conditions under which switch from Th1 to Th2 immune response occurs during MAP infection. We specifically consider two hypotheses: 1) switch is driven by accumulation of extracellular bacteria that in turn skew differentiation towards the Th2 response, and 2) switch is caused by exhaustion/suppression of Th1 response and concomitant rise of Th2 response. We investigate the conditions under which these mathematical models give rise to the Th1 to Th2 switch. We show that the following factors strongly influence Th1/Th2 switching dynamics: the mechanism by which MAP-specific Th cells are maintained at the site of infection (continuous differentiation from naïve T cells or local proliferation), rate at which Th1 response is exhausted, longevity of extracellular bacteria, and the efficiency at which immune responses cross regulate each other.

## Model

**Figure 2:**
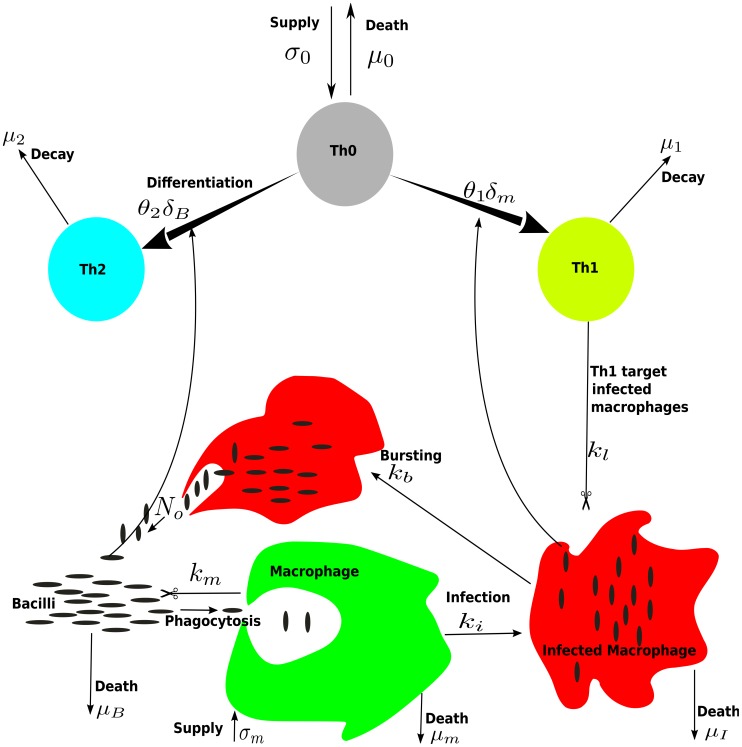
Cartoon illustrating interactions between the bacteria, macrophages, and CD4 T cells occurring during MAP infection. In the basic model, extracellular bacteria infect macrophages, and infected macrophages release bacteria upon cell death. Th1 and Th2 cell responses are generated from naïve T cells, Th0, depending on the amount of infected macrophages and free bacteria, respectively. In this model populations of Th1 and Th2 cells are maintained only by the constant differentiation and expansion of the MAP-specific Th0 cell population into Th1/Th2 subsets. Thick arrows represent Th0 cell differentiation and clonal expansion into the Th1 and Th2 subsets.

To study factors that may contribute to the dynamics of MAP and MAP-specific Th1 and Th2 responses we propose a novel mathematical model. The model is based on the current biological understanding of basic properties of mycobacterial infections [20], [21], [46], and experimental and theoretical understanding of Th1 and Th2 effector differentiation from naïve T cells [28], [30], [33], [38] (*see* introduction). The model includes interactions between extracellular MAP bacteria (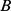), macrophages (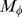) (target cells), naïve CD4 T cells (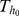), and the two subsets of the MAP-specific immune response, Th1 (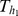) and Th2 (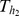) cells (Figure 2). Infection is initiated by extracellular bacteria at the dose 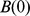. Macrophages internalise extracellular bacteria and get infected at a rate 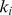 giving rise to infected macrophages (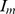). There is still uncertainty in the literature on how macrophages are maintained at local sites such as the gut [47]. In the model we assume that during infection, macrophages are supplied from progenitor monocytes that are recruited from the blood to the site of infection at a rate 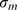. Infected macrophages burst at a rate 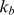 releasing 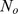 bacteria into the extracellular environment. Th1 effectors remove infected macrophages at a rate 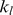 and intracellular bacteria are killed in this process. Effector CD4 T cells activate macrophages to kill intracellular bacteria and help with the generation of the MAP-specific CD8 T cell response which in turn clears infected macrophages [48]. Extracellular bacteria are cleared at a rate 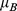. Some extracellular bacteria are taken up by macrophages and are destroyed at a rate 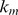. Furthermore, given available experimental data [24] we assume that MAP-specific antibodies (Th2 response) are ineffective at eliminating extracellular bacteria. Uninfected and infected macrophages have death rates of 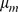 and 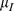, respectively.

Selective differentiation of naïve CD4 T cells into either Th1 or Th2 effectors is established during priming caused by interaction of major histocompatibility complex (MHC)-specific peptide complexes on antigen-presenting cells and T-cell receptors [25], [26]. It is generally believed that Th1 responses are generated against intracellular pathogens such as viruses while Th2 responses are generated against extracellular pathogens such as extracellular bacteria [40], [49]–[51]. Factors that influence priming and differentiation of CD4 T cells include the dose and type of antigen, co-stimulatory molecules and/or antigen presenting cells, and the cytokine environment present during priming [25], [38]. In our model, MAP-specific naïve CD4 T cells (Th0) are produced at a rate 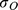 continuously from the thymus [52] and decay at rate 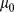. Th0 cells are recruited into the Th1 and Th2 immune responses at per capita rates 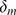 and 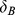, respectively. Recruitment rates depend on the density of infected macrophages and extracellular bacteria. We make the simplest assumption that Th1 response is driven by the density of infected macrophages 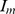, and Th2 response is driven by the density of extracellular bacteria 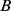. Following recruitment, naïve CD4 T cells undergo a program of division and differentiation resulting in a large population of MAP-specific effectors. Therefore, in the model Th1 effectors are produced at a rate 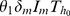 and Th2 effectors are produced at a rate 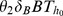 where 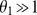 and 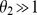 are the parameters determining the magnitude of clonal expansion of the Th1 and Th2 responses, respectively. Th1 and Th2 effectors decay at rates 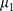 and 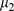, respectively. With these assumptions (Figure 2) the basic mathematical model is given by the following system of differential equations (*see* the Supplemental Information (Text S1) for the basic properties of the model and derivation of the basic model reproduction number, 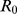):
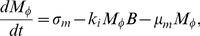
(1)
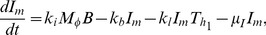
(2)
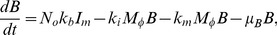
(3)
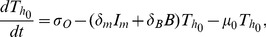
(4)
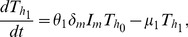
(5)
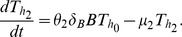
(6)

Very little quantitative detail regarding MAP infection of ruminants is available, and therefore, most of the parameters of our mathematical model are unknown. Information on immunological responses to MAP infection was collected from different ruminant species when there was no strong contradictory findings among the species. Thus, our model is not for a particular ruminant. We used several different strategies to provide some estimates for the model parameters. Some parameters have been estimated from published experimental data (Table 1). Also, we have carried out literature search to obtain general acceptable ranges for other parameters. Well established biological knowledge was used to estimate cell populations in the sheep gut (Table 2). Where information about a parameter was not found, estimates were used that enable observation of cell kinetics within an acceptable range of the estimated cell population per 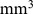 in the gut. Yet, it still should be emphasized that most of our parameters are only educated guesses. Therefore, to determine which parameters in the model impact the most the time of the Th1 to Th2 switch we also performed standard parameter sensitivity analysis [53].

**Table 1:** Model parameters.

Name	Definition	Dimension	Range	Value
	Parameters that were fixed in model fitting			
*σ_m_*	Macrophage supply	cell mm^−3^ day^−1^	8.0–10.0	10.0
*σ_O_*	Th0 supply	cell mm^−3^ day^−1^	0.00001–0.001	0.001
*μ_m_*	Macrophages death rate	day^−1^	0.11–0.025	0.02
*μ_I_*	Infected macrophages death rate	day^−1^	0.11–0.025	0.02
*μ_B_*	Bacteria death rate	day^−1^	0–1.0	0.03
*μ*_0_	Th0 decay/death rate	day^−1^	0.1–0.01	0.01
*μ*_1_	Th1 decay/death rate	day^−1^	0.1–0.01	0.03
*μ*_2_	Th2 decay/death rate	day^−1^	0.01–0.01	0.02
*k_m_*	Bacteria removal by macrophages	mm^−3^ day^−1^	0–10^−4^	0.000125
*k_l_*	Th1 lytic effect	mm^−3^ day^−1^	0.0–0.2	0.00004
*N_o_*	Burst size		80.0–100.0	100.0
	Parameters that were varied in model fitting			
*θ*_1_	Th1 cells clonal expansion	-	1.0−10^4^	9000.0
*θ*_2_	Th2 cells clonal expansion	-	1.0−10^4^	9000.0
*δ_m_*	Th0 differentiation into Th1 cells	mm^−3^ day^−1^	0.0–1.0	0.01
*δ_B_*	Th0 differentiation into Th2 cells	mm^−3^ day^−1^	0.0–1.0	0.01
*k_i_*	Macrophage infection rate	mm^−3^ day^−1^	0–10^−2^	0.002
*k_b_*	Infected macrophages burst rate	day^−1^	0–10^−4^	0.00075

We list parameters that have been derived using information from the following literature sources [12], [30], [65]–[69] and are considered as the default values in the model simulations. Fixed parameters were not changed in the simulations and in the model fitting to data, while the rest were allowed to vary.

**Table 2:** Estimates of cell measurements in the sheep gut used in the model.

Cell volume measurements	Estimated parameter
B-cells∼2×10^9^ cells/litre	∼2×10^3^ cells/mm^3^
T-cells∼1×10^9^ cells/litre	∼1×10^3^ cells/mm^3^
Monocytes∼4×10^8^ cells/litre	∼4×10^2^ cells/mm^3^
Small intestines ∼10 litres	
Bacteria oral inoculation ∼1×10^8^ in gut	∼0.1/mm^3^
Bacteria shedding ∼1×10^3^/*mm*^3^	
Th0 cells are∼1 in 10^6^ of CD4 cells	∼0.00001–0.001/mm^3^

Cells	Half-life
Macrophages	6–30 days
B cells	5–6 weeks
T cells	1–3 weeks

Initial conditions	Estimation
Macrophages, *M_φ_*(0) ∼4–5×10^2^	∼400–500/mm^3^
Bacteria, *B*(0)	∼0.1/mm^3^
Th0, *T_h_*_0_(0)	∼0.1/mm^3^

**Table 3:** Parameter estimates explaining the kinetics of Th1 and Th2 responses in sheep experimentally infected with MAP.

Th1/Th2 data	*R*_0_	*B*(0)	*k_i_*	*k_b_*	*δ_m_*	*δ_B_*	*θ*_1_	*θ*_2_	RSS
Classical switch	4.4	1.8×10^−5^	0.9786	0.00093	-	0.9998	-	-	0.038
Delayed switch	3.3	1.7×10^−8^	-	-	0.00072	-	14000	25000	0.0078
Combined response	2.8	6.2×10^−2^	0.000598	-	-	-	12529	18581	0.089
Th1 only response	3.7	8.8×10^−8^	-	0.00083	-	-	5798	321	0.001

The basic model (Eqns. (1)–(6)) was fitted to the normalised IFN-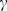 (Th1 response) and antibody titers (Th2 response) data [20]. Dash “-” indicates that this parameter was fixed to its default value given in Table 1. Parameters 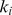, 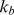, 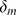, 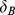 were constrained between 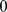 and 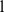, while 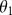 and 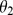 were constrained between 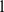 and 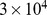. Estimated 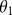 and 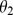 values for the delayed switch and combined Th1/Th2 response data are a bit higher though reasonably comparable to the magnitude of naïve CD8+ T cell expansion estimated in studies [70], [71] to be in the order of 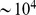 and 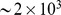 in mouse spleen. RSS stands for the residual sum of squares.

## Results

### Model behavior

**Figure 3:**
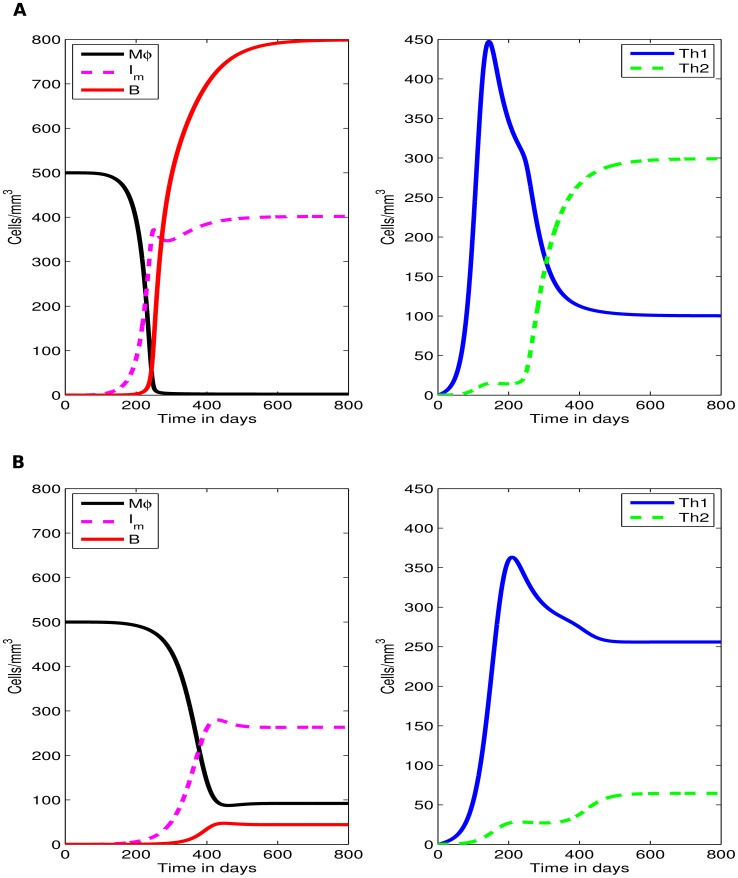
Longevity of MAP in extracellular environment impacts the kinetics of Th1/Th2 switch. We simulate dynamics of the uninfected and infected macrophages and extracellular bacteria (left panels) and MAP-specific Th1 and Th2 responses (right panels) for the set of parameters shown in Tables 1&2. When extracellular bacteria are long lived (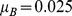 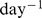), the switch from Th1 to Th2 response naturally emergence in MAP-infected animals (panel **A**). However, when extracellular bacteria are relatively short lived (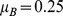 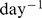), Th1 to Th2 switch is not observed (panel **B**). Additional parameters are 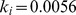 cell mm^−3^ 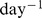 (panel **A**) and 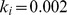 cell 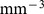 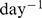 (panel **B**). Infection rate 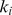 was changed in panel **B** to keep the basic model disease reproduction number, 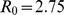, the same for both simulations.

From many experiments it is well known that Th1 and Th2 responses cross-inhibit each other [28], [38], [46], [54]; in particular Th1 cytokines generally suppress differentiation of naïve CD4 T cells into Th2 effectors and *vice versa* [30], [38], [55], [56]. However, most of the information on cross-inhibition comes from *in vitro* studies under strong polarising conditions and it remains unknown if such cross-inhibition also occurs during infections *in vivo*. In our basic mathematical model we neglected the possibility of cross-inhibition and investigated whether Th1/Th2 switch could be achieved if Th1 and Th2 responses are not directly cross-suppressive. Surprisingly, this model was able to predict accumulation and loss of the Th1 response and thus the switch from the dominant Th1 to dominant Th2 response during the infection (Figure 3A).

In the model, the phenomenon occurs due to the following steps. Due to high infectivity of free bacteria and a large population of resident macrophages, many macrophages become infected and very few extracellular bacteria exist. The large population of infected macrophages leads to generation of a Th1 response which, however, lacks the ability to eliminate the infection [57], [58]. Infected macrophages produce new bacteria which in turn infect newly arriving macrophages. A quasi equilibrium is established. Because Th1 response is unable to clear extracellular bacteria and because in this simulation extracellular bacteria are relatively long lived (Table 1), extracellular bacteria accumulate over time. The increase in free bacteria then skews differentiation of Th0 cells toward Th2 phenotype, and this process indirectly suppresses generation of Th1 response which starts to decline over time. Therefore, the two assumptions are sufficient to drive the switch of the initially dominant cellular (Th1) response to antibody (Th2) production. These assumptions are 1) the generation of Th1 response is driven by density of infected macrophages while the generation of the Th2 response is driven by free bacteria (see Eqns. (1)–(6)), and 2) extracellular bacteria are long lived. Indeed, increasing the death rate of extracellular bacteria (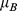) effectively removes the Th1/Th2 switch whereby both responses are able to persist and Th1 response remains dominant (Figure 3B). This occurs because if extracellular bacteria are cleared rapidly, density of the bacteria remains proportional to the density of macrophages and thus, Th2 response never outgrows the initially dominant Th1 response.

Further insights into the dynamics of the Th1/Th2 switch can be obtained by calculating the dynamics of the ratio of the density of Th1 to Th2 response, 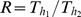 (*see* the Derivation of the Th1/Th2 ratio equation Section in the Supplemental Information (Text S1) for the derivation of the equation for 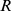 dynamics). The dynamics of the ratio in the model is given by
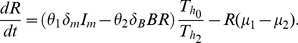
(7)

When 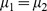 under assumption of a quasi steady state for 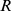 (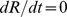) we find that 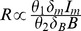, and therefore for the ratio to slowly change over time, the ratio of infected macrophages to free bacteria should change from a value more than one to a value less than one. This in general occurs when extracellular bacteria are long lived outside of macrophages [59] and the number of bacteria released per infected macrophage is relatively low [60]. However, Eqn. (7) also shows that if the decay rate of the Th1 response is much greater than that of Th2 cells (i.e., 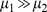), the Th1/Th2 switch may still occur at high rates of clearance of extracellular bacteria (results not shown).

### Comparing model simulations to experimental data

Experimental infections of sheep with MAP showed four different patterns of the immune response development: the so-called classical Th1/Th2 switch (Figure 1A), delayed Th1/Th2 switch (Figure 1B), a combined Th1/Th2 response (Figure 1C) and a Th1 only response (Figure 1D) [20]. We investigated whether our basic mathematical model can reproduce these experimental patterns. To compare model predictions with experimental data, we normalised the predicted Th1 and Th2 response by their maximum value reached in infection. Note that this is different from the Th1/Th2 dynamics shown in Figure 3 where cell populations are not normalised. To avoid over-fitting, we selected parameters for fitting the data predicted as most important for determining timing of Th1/Th2 switch using sensitivity analysis (Figure S1 and the sensitivity analysis Sections in the Supplemental Information (Text S1)). These include parameters that drive infection dynamics (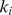, 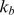) and parameters that control differentiation and recruitment of effector T cells (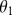, 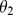, 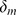, and 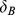). The least squares method was employed using the *patternsearch* function in MATLAB.

**Figure 4:**
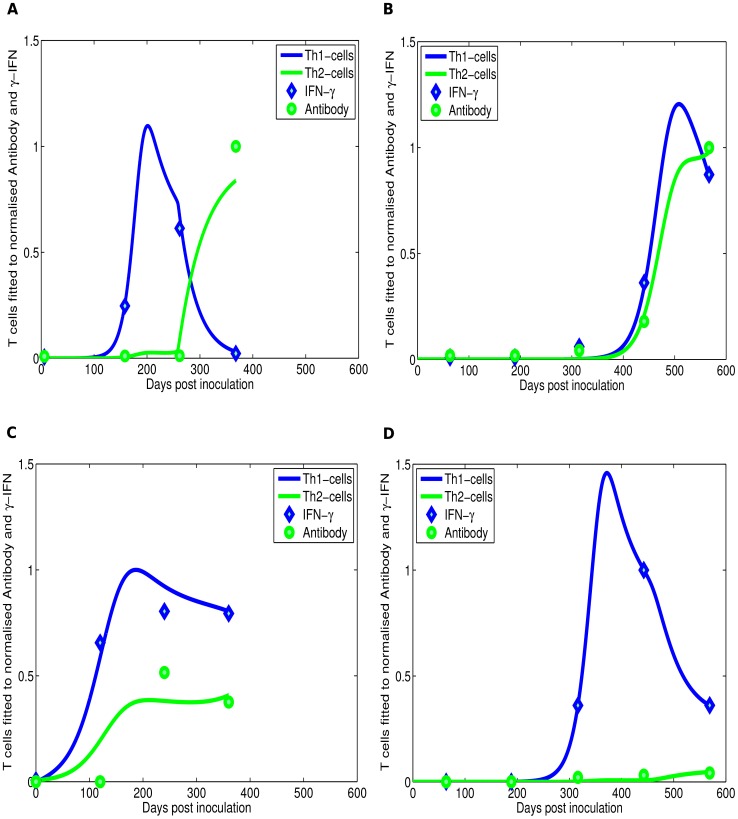
Model fits of experimental infection data. The basic mathematical model (Eqns. (1)–(6)) explains the major patterns of dynamics of the MAP-specific Th1 (IFN-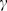) and Th2 (antibody) responses in MAP-infected sheep ([20], see Figure 1). Lines are predictions of the mathematical model fitted to the normalised experimental data. Panel **A** shows the data and model prediction of the classical Th1/Th2 switch, panel **B** shows the Th1/Th2 delayed switch, panel **C** shows the combined Th1/Th2 response, and panel **D** shows only Th1 response (Figure 1). Fixed parameters are given in Table 1 and estimated parameters are given in Table 3.

As our results show, the model can relatively well reproduce all major patterns of Th1/Th2 dynamics in MAP-infected sheep (Figure 4). The major parameters that determine the type of the response is the initial bacterial dose and parameters determining the kinetics of the immune response.

### Effect of initial dose size on the switch time

**Figure 5:**
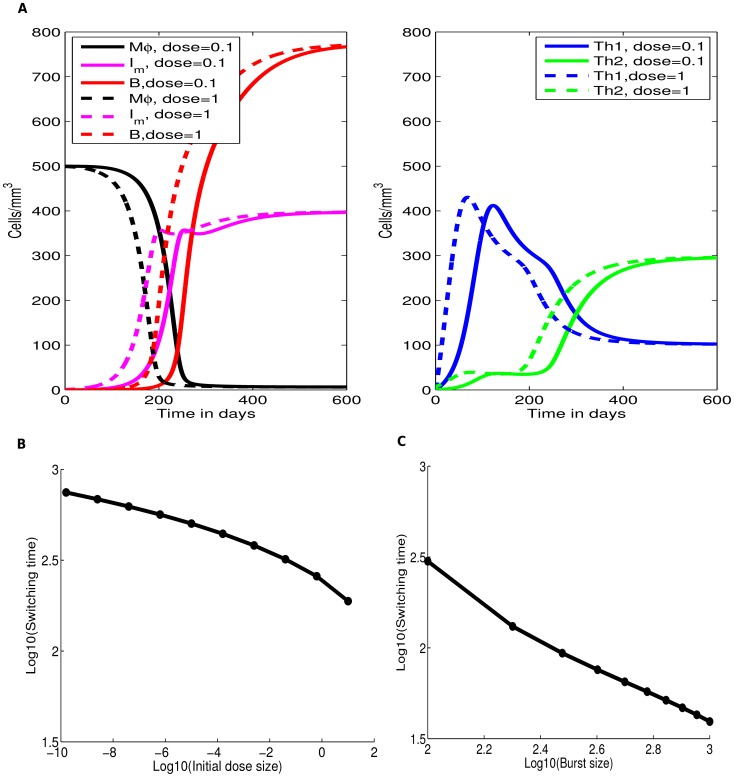
Increasing the initial bacterial dose 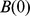 and virulence of the bacteria (burst size 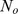) reduces the time of the Th1/Th2 switch. We use the basic mathematical model (Eqns. (1)–(6)) to simulate infection and immune response dynamics at different initial bacterial doses 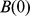 (panels **A** and **B**) or at different numbers of bacteria released per infected macrophage 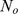 (panel **C**). In panel **A** we show the dynamics of uninfected (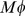) and infected (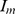) macrophages and extracellular bacteria (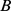). Parameters used for the simulations are given in Table 1.

Timing of the Th1 and Th2 switch was further investigated by varying the initial bacterial inoculation dose 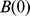 and the burst size of infected macrophages 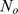 (Figure 5). Both of these parameters can be manipulated experimentally. Increasing the initial bacterial dose resulted in a faster switch, but a very large increase in the dose is needed to observe a noticeable decrease in the switch times (Figure 5A&B). Increasing the dose size results in more macrophages being initially infected leading to a rapid depletion of uninfected macrophages and generation of the Th1 immune response. Rapid growth of the population of infected macrophages leads to accumulation of extracellular bacteria. Early and rapid growth of the bacterial population pushes for early Th2 immune response development, resulting in an early Th1/Th2 switch. Importantly, increasing the burst size 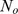 is more effective at reducing the time of the Th1/Th2 switch than equivalent increase in the initial bacterial dose (Figure 5C). Both of these predictions can be tested by infecting animals with different initial doses or with MAP strains that differ in virulence.

## Discussion

Progression of MAP infection in ruminants often occurs concomitantly with a switch from dominance of the MAP-specific Th1 immune response to a dominance of a Th2 response. Previous studies have shown that animals with paucibacillary lesions are likely to express a cell mediated, Th1-type, immune response that is protective against intracellular bacteria, while a Th2-type response is generally detected in animals with multi-bacillary lesions [18]–[20]. In this study, we have developed the first mathematical model of the helper T cell response to MAP and have analysed mechanisms that influence the dynamics of Th1 to Th2 switch during disease progression in MAP-infected animals. A number of interesting results emerged from the analysis of the model.

First, the model is able to simulate two main infection outcome scenarios, (i) elimination of infection, this is associated with an initial strong Th1 immune response (Th1 only response), (ii) infection persistence (or latency), this is marked by both a Th1 and a Th2 response with high expression of a Th1 response over a Th2 response (classical and delayed switch). Second, if the extracellular bacteria are not readily removed by the host's innate immune system, simulations show a Th1/Th2 switch which is characterized by accumulation of long-lived extracellular bacteria. Third and finally, the basic model was able to explain different patterns of the dynamics of MAP-specific Th1 and Th2 responses as was observed in experimental infections of sheep (Figures 1 and 4). These results from the basic model suggest that Th1/Th2 switch may be a result of disease progression rather than the cause.

We find that in our basic mathematical model the longevity of extracellular bacteria is one of the key factors driving Th1/Th2 switch and disease progression in MAP-infected animals (Figure 3). Long survival of extracellular bacteria also naturally explains increased shedding of bacteria in animals with JD. Of note, extended models that assume a relatively short survival time of extracellular bacteria do not predict accumulation of MAP over the course of infection (*see* Section Alternative models in the Supplemental Information (Text S1)). Estimation of the average survival time of MAP in extracellular environment in the host will allow further refinement of our mathematical model. Such measurements may also suggest which additional mechanisms need to be involved to explain kinetics of Th1/Th2 responses in MAP-infected animals.

Several other mechanisms may influence the likelihood and the kinetics of the Th1/Th2 switch including inhibition of Th0 cell differentiation by Th1 and Th2 responses, proliferation of effector T cells at the site of infection, and exhaustion of protective Th1 responses due to exposure to the antigen (*see* Section Alternative models in the Supplemental Information (Text S1)). The first two mechanisms, differentiation inhibition and local proliferation can dramatically alter the course of MAP-specific Th1 and Th2 responses depending on the strength of inhibition and sensitivity of Th1/Th2 cells to the local antigen concentrations. Therefore, future experimental studies should focus more on these dynamic processes and determine whether they occur in MAP-infected animals.

Interestingly, we found that inhibition of effector functions of Th1 cells by Th2 response (and *vice versa*) did not influence the kinetics of the Th1/Th2 switch in the basic mathematical model when clearance rate of extracellular bacteria is high (results not shown). In part, this is because in the basic model the Th1/Th2 switch is driven by the accumulation of extracellular bacteria and reduction of the efficacy of MAP-specific Th1 cells does not influence bacterial loads, and therefore, does not impact the kinetics of the Th1/Th2 switch.

Although our mathematical model considers a number of important biological processes and illustrates the wealth of different scenarios for the dynamics of MAP-specific Th1 and Th2 responses, several potentially important biological details have been ignored in the model. First, we did not consider the dynamics of MAP-specific Th17 cells and regulatory T cells which are thought to play important role during intestinal infections in mice and humans [61]. There is, however, limited evidence that these subsets play a critical role in control of MAP replication but more experimental data in this area need to be collected. Secondly, in our mathematical model we ignored so-called “cellular plasticity” of effector CD4 T cell responses where effectors with a particular phenotype (e.g., Th1) may potentially convert into effectors of another phenotype (e.g., Th2) [62]. Exact mechanisms of how such cellular plasticity is regulated especially during chronic infections are still unknown. In our mathematical model we assumed only “population plasticity” where the change in the phenotype of MAP-specific T cells occurs due to differences in the rates of differentiation, proliferation and death at the site of infection [62]. Additional experimental studies will be needed to determine if conversion of protective MAP-specific Th1 cells into detrimental Th2 cells actually occurs during MAP infection.

Thirdly, to keep the model simple, we only captured the role of Th1 cells (CD4 T cells) to represent the cell mediated response. There is evidence of accumulation of CD8 T cells in MAP infection [63], which also secrete IFN-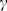 and can lyse infected macrophages. However, whether their involvement contribute significantly in the control of MAP is yet to be clearly demonstrated. Mycobacteria mainly resides within vacuoles of infected cells and therefore most of bacterial antigens are presented on MHC-II molecules (which are recognised by CD4 T cells). It was postulated in the study of Chiodini and Davis [64], that CD8+ T cells may be key in the development of the protective immunity through modulating the regulatory activity of 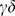 T cells, although the exact mechanism of this cooperation was not presented.

Also, in this study we did not include the role of M-cells and enterocytes, which are essential in the establishment of MAP infection as entry vehicles to pass the bacteria to professional phagocytes (mucosal macrophages). There is potential to understand better the host-pathogen immune interactions using a spatial model that captures pathogen and immune response components (both innate and adaptive) at different locations and stages of disease. However, currently there is limited quantitative data to parameterise a spatial compartmentalised model. Furthermore, infection progression and priming of the adaptive immune response is mostly centred on macrophages, which (i) have the capacity to stimulate the immune response and (ii) harbor MAP for a long time period. Apart from phagocytic properties, macrophages also present antigens to CD4+ cells in the context of MHC-II molecules. Hence, the main modeling assumptions used in this current study and the choice of a simple model that does not include different compartments.

Our study suggests that Th1/Th2 switch in MAP infection can be explained through (i) different regulation of Th cell differentiation, (ii) bacteria accumulation, (iii) proliferation and differentiation inhibition of T cells, and (iv) Th1 immune exhaustion. Some of these results are echoed in previous mathematical modeling studies. For instance, the studies [34], [35] showed that when effectors fail to clear the antigen, the initially dominant Th1 response is lost and Th2 response arises. The studies [31], [33], [37]–[39] showed that cytokines that are present at the site of infection can influence the direction of naïve CD4 T cell differentiation, and therefore may determine the shift in the effector dominance. This mechanism was shown to be regulated by specific transcription factors such as T-bet, GATA-3, FoxP3 and ROR-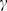
t which directs differentiation of naïve T cells into specific effector subsets [62]. It should be noted that previous mathematical models of Th1/Th2 regulation focused on cross regulation of Th1/Th2 cell responses based on cell to cell interactions via Th1/Th2 cytokines [34]–[36], [41]. To the best of our knowledge our study is the first to model Th1/Th2 dynamics in MAP infection (*see* the discussion for Th1/Th2 insights from previous mathematical studies).

In summary, our JD immunology models indicated that the following factors can determine the timing of Th1/Th2 switch: (i) bacteria dose size and the burst size of infected macrophages, (ii) longevity of extracellular bacteria, (iii) degree of competition between Th1 and Th2 responses, and (iv) Th1 immune exhaustion. Testing these model predictions using more detailed experimental data that can be obtained from infecting animals with (i) MAP-strains of different virulence, (ii) different initial doses of MAP, and (iii) measuring the fraction of intracellular vs. extracellular MAP in tissues *in vivo* will help identify the mechanism controlling the kinetics of Th1/Th2 immune responses. JD is associated with slow disease progession in cattle, therefore experiments using small ruminants such as sheep and goats that are associted with relatively fast disease progression are recommended to make the experiments less expensive and to allow for reasonable time to collect the required data.

## Supporting Information

Figure S1**Sensitivity analysis of the model parameters.** Using latin hypercube sampling (LHS) of parameters and partial rank correlation coefficients (PRCCs) we determined the influence of various model parameters to the time of the switch from Th1 to Th2 response in the basic mathematical model (Eqns. (1)–(6)). We list only parameters that significantly influence Th1/Th2 switch time at 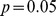. The 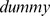 parameter shows the level of significance of a parameter with little influence in the model dynamics indicating that model parameters with such low PRCCs do not significantly alter the output variable. Parameter values and ranges used in LHS (a uniform distribution was used to draw 200 samples for each parameter) are given in Table 1. Negative PRCCs values indicate parameters increase which reduces the time of the Th1/Th2 switch, and positive PRCCs values indicate parameters increase which increases the time of the Th1/Th2 switch.(EPS)

Figure S2 **Cartoon illustrating interactions between the bacteria, macrophages, and CD4 T cells occurring during MAP infection in the extended model.** Extracellular bacteria infect macrophages, and infected macrophages release bacteria upon cell death. Th1 and Th2 cell responses are generated from naïve T cells, Th0, depending on the amount of infected macrophages and free bacteria, respectively. Populations of Th1 and Th2 cells are maintained by differentiation, expansion of the MAP-specific Th0 cell population into Th1/Th2 subsets, and by local proliferation of Th1/Th2 effectors. T cell population can be affected by the cross-inhibition of the Th1/Th2 responses at the level of the differentiation of Th0 cells into Th1 or Th2 effectors and effector function of Th1/Th2 responses, and through the exhaustion of MAP-specific Th1 effectors due to chronic antigenic stimulation. Thick arrows represent Th0 cell differentiation and clonal expansion into the Th1 and Th2 subsets.
(EPS)

Figure S3**MAP infection and immune response kinetics simulated with alternative mechanisms.** Several additional mechanisms can drive the Th1/Th2 switch in MAP-infected animals when extracellular bacteria are cleared rapidly. We use parameters as in Figure 3B (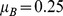 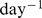 and 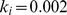 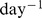) which do not lead to the Th1/Th2 switch and add alternative terms to the basic model (see Alternative models Section). Panel **A** shows the dynamics of the infection when there is inhibition of differentiation of naïve CD4 T cells into Th1 response by Th2 cells (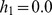 and 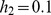 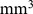/cell). Panel **B** shows the dynamics of infection when there proliferation of effector Th1 and Th2 responses (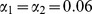 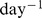, 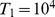 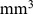/cell, and 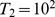 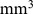/cell). Panel **C** shows the dynamics of infection when the MAP-specific Th1 response becomes exhausted over time (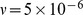 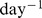).(EPS)

Text S1 **Supplemental information.** Mathematical anaylsis of the model, sensitivity analysis of model parameters, and alternative model results.
(PDF)
