# Supplementary material for: Competition for Antigen between Th1 and Th2 Responses Determines the Timing of the Immune Response Switch during Mycobaterium avium Subspecies paratuberulosis Infection in Ruminants
Source: PLoS Comput Biol. 2014 Jan 9;10(1):e1003414. doi: 10.1371/journal.pcbi.1003414 (PMC3886887; doi:10.1371/journal.pcbi.1003414)
Supplement: Text S1 — Supplemental information. Mathematical anaylsis of the model, sensitivity analysis of model parameters, and alternative model results. (PDF) [file pcbi.1003414.s004.pdf]

Competition for antigen between Th1 and Th2 responses determines the timing of the immune response switch during *Mycobacterium avium* subspecies *paratuberculosis* infection in ruminants

Gesham Magombedze[1,*], Shigetoshi Eda[2], and Vitaly V. Ganusov[1,3,4]

[1]National Institute for Mathematical and Biological Synthesis,
[2]Department of Forestry, Wildlife, and Fisheries,
[3]Department of Microbiology,
[4]Department of Mathematics,
University of Tennessee, Knoxville, TN 37996-1527 USA

∗ E-mail: gmagombedze@nimbios.org;gmagombedze@gmail.com

Supplemental Information

Model analysis

Starting with non-negative initial conditions the structure of the model gives non-negative solutions. The model disease free state (DFE), E_0 is given by

$$E_0 = (\hat{M}_\phi, \hat{I}_m, \hat{B}, \hat{T}_{h_0}, \hat{T}_{h_1}, \hat{T}_{h_2}) = (\frac{\sigma_m}{\mu_m}, 0, 0, \frac{\sigma_0}{\mu_0}, 0, 0).$$

The model disease equilibrium state (DEE), E_1 (we could not express the DEE in a closed form) is given by

$$E_1 = (\bar{M}_\phi, \bar{I}_m, \bar{B}, \bar{T}_{h_0}, \bar{T}_{h_1}, \bar{T}_{h_2}).$$

Computation of the basic model disease reproduction number (R_0)

The basic model disease reproduction number was obtained using the next generation method by Castillo-Chavez *et al* [1]. The basic model can be written in the form:

$$\begin{aligned} \frac{dX}{dt} &= f(X, Y, Z), \\ \frac{dY}{dt} &= g(X, Y, Z), \\ \frac{dZ}{dt} &= h(X, Y, Z), \end{aligned}$$

where $X = (M\phi, T_{h_0}, T_{h_1}, T_{h_2}) \in \Re^4$, $Y = I_m \in \Re$ and $Z = B \in \Re$ and $h(X, 0, 0) = 0$. X denote uninfected macrophages and T cell sub-types, Y denotes infected macrophages and Z denotes free

bacteria (the pathogen causing infection), therefore $E_0 = (X^*, 0, 0)$. Assuming that $\tilde{g}(X^*, Y, Z) = 0$ implicitly determines a function $Y = \tilde{g}(X^*, Y)$. Let $A = D_Z h(X^*, \tilde{g}(X^*, 0), 0)$ and further assume that A can be written in the form $A = M - D$, with $M \geq 0$ (that is $m_{ij} \geq 0$) and $D > 0$, a diagonal matrix. Then, the basic model disease reproduction number is defined as the spectral radius (dominant eigenvalue) of the matrix MD^{-1}, that is $R_0 = \rho(MD^{-1})$.

Now, with $X = (M\phi, T_{h_0}, T_{h_1}, T_{h_2})$, $Y = I_m$, and $Z = B$, we evaluate that

$$\tilde{g}(X^*, Y) = \frac{k_i M\phi B}{(k_b + k_l T_{h_1} + \mu_I)} \quad \text{and} \quad h(X^*, \tilde{g}(X^*, Y), Z) = \frac{N_o k_b k_i M\phi B}{(k_b + k_l T_{h_1} + \mu_I)} - B(k_i M\phi + k_m M\phi + \mu_B).$$

Therefore, $A = D_Z h(X^*, \tilde{g}(X^*, 0), 0) = \dfrac{N_o k_b k_i \hat{M}\phi}{(k_b + \mu_I)} - (k_i \hat{M}\phi + k_m \hat{M}\phi + \mu_B)$. Hence $M = \dfrac{N_o k_b k_i \hat{M}\phi}{(k_b + \mu_I)}$, $D = (k_i \hat{M}\phi + k_m \hat{M}\phi + \mu_B)$ and

$$\begin{aligned} R_0 &= MD^{-1}, \\ &= \frac{k_b k_i \hat{M}\phi N_o}{(k_i \hat{M}\phi + k_m \hat{M}\phi + \mu_B)(k_b + \mu_I)}, \\ &= \frac{k_b k_i \sigma_m N_o}{\Big(\sigma_m(k_i + k_m) + \mu_m \mu_B\Big)\Big(k_b + \mu_I\Big)}. \end{aligned} \quad \text{(S.1)}$$

Theorem 1. *E_0 is locally asymptotically stable if $R_0 < 1$ and unstable if $R_0 > 1$.*

Proof of local stability of E_0:
The Jacobian matrix of the system of equations (1)-(6) (which represent the basic model) evaluated at the DFE is

$$J = \begin{bmatrix} -\mu_m & 0 & -k_i \hat{M}\phi & 0 & 0 & 0 \\ 0 & -(k_b + \mu_I) & 0 & 0 & 0 & \\ 0 & N_o k_b & -(k_i \hat{M}\phi + k_m \hat{M}\phi + \mu_B) & 0 & 0 & 0 \\ 0 & -\delta_m \hat{T}_{h_0} & -\delta_B \hat{T}_{h_0} & -\mu_0 & 0 & 0 \\ 0 & \theta_1 \delta_m \hat{T}_{h_0} & 0 & 0 & -\mu_1 & 0 \\ 0 & 0 & \theta_2 \delta_B \hat{T}_{h_0} & 0 & 0 & -\mu_2 \end{bmatrix}.$$

The eigenvalues of the Jacobian matrix, J, can be determined by solving the characteristic equation $|J - \lambda I| = 0$, which yeilds the following eigenvalues $\lambda_1 = -\mu_m$, $\lambda_2 = -\mu_0$, $\lambda_3 = -\mu_1$, $\lambda_4 = -\mu_2$, and the polynomial

$$\lambda^2 + \lambda\Big((k_b + \mu_I) + (k_i \hat{M}\phi + k_m \hat{M}\phi + \mu_B)\Big) + (k_b + \mu_I)(k_i \hat{M}\phi + k_m \hat{M}\phi + \mu_B) - k_i \hat{M}\phi k_b N_o = 0 \quad \text{(S.2)}$$

With $\lambda_{1,2,3,4} < 0$, to complete the proof for local stability we apply the Routh Hurwitz criterion, which requires that, in polynomial equation (S.2)

$$\begin{aligned} &\text{(i)} \quad (k_b + \mu_I) + (k_i \hat{M}\phi + k_m \hat{M}\phi + \mu_B) > 0, \\ &\text{(ii)} \quad (k_b + \mu_I)(k_i \hat{M}\phi + k_m \hat{M}\phi + \mu_B) - k_i \hat{M}\phi k_b N_o > 0. \end{aligned}$$

These conditions are satisfied if $(k_b + \mu_I)(k_i \hat{M}\phi + k_m \hat{M}\phi + \mu_B) > k_i \hat{M}\phi k_b N_o$, that is if

$$1 > \frac{k_b k_i \hat{M}\phi N_o}{(k_i \hat{M}\phi + k_m \hat{M}\phi + \mu_B)(k_b + \mu_I)},$$

hence $1 > R_0$, therefore $R_0 < 1$. Thus, the DFE is locally asymptotically stable $\square$

Global stability of E_0

Using the method by Castillo-Chavez *et al.* [1] to establish the global stability of the DFE.

Set $X = (M_\phi, T_{h_0}, T_{h_1}, T_{h_2})$ and $Z = (I_m, B)$. The model equations are rewritten as follows:

$$\begin{aligned}\dot{X} &= F(X, 0),\\ \dot{Z} &= G(X, Z).\end{aligned}$$

Theorem 2. *The DFE is globally asymptotically stable when $R_0 < 1$, if (i) $E_0 = (X^*, 0)$ is locally asymptotically stable and (ii) $\hat{G}(X, Z) = AZ - G(X, Z) \geq 0$ in the biological feasible region, where $A = D_Z(X^*, 0)$ is the Jacobian of $G(X, Z)$ evaluated at $(X^*, 0)$.*

Proof of global stability of E_0:
Therefore, Condition (ii)

$$F(X, 0) = \begin{bmatrix} \sigma_m - \mu_m M_\phi \\ \sigma_0 - \mu_m T_{h_0} \\ 0 \\ 0 \end{bmatrix}, \quad \text{and} \quad \hat{G}(X, Z) = \begin{bmatrix} k_l \bar{T}_{hi} \\ 0 \end{bmatrix} \geq 0.$$

Therefore, $\hat{G}(X, Z) \geq 0$. Condition (i) follows from *Theorem: 1*. Thus, the DFE is globally asymptotically stable □

Derivation of the Th1/Th2 ratio equation

We define the ratio, R, to be given by $\frac{T_{h_1}}{T_{h_2}}$ $(R = \frac{T_{h_1}}{T_{h_2}})$. Defferentiating R with respect to time gives

$$\frac{dR}{dt} = \Big(\frac{1}{T_{h_2}}\Big)\dot{T}_{h_1} - \Big(\frac{T_{h_1}}{T_{h_2}^2}\Big)\dot{T}_{h_2}.$$

Substituting $\dot{T}_{h_1}$ and $\dot{T}_{h_2}$ with the differential equation representing the time kinetics of Th1 cells and Th2 cells, respectively, gives

$$\begin{aligned}\frac{dR}{dt} &= \frac{1}{T_{h_2}}\Big(\dot{T}_{h_1} - (\frac{T_{h_1}}{T_{h_2}})\dot{T}_{h_2}\Big),\\ &= \frac{1}{T_{h_2}}\Big(\theta_1\delta_m I_m T_{h_0} - \mu_1 T_{h_1}\Big) - \frac{T_{h_1}}{T_{h_2}^2}\Big(\theta_2\delta_B T_{h_0} - \mu_2 T_{h_2}\Big),\\ &= \theta_1\delta_m I_m\Big(\frac{T_{h_0}}{T_{h_2}}\Big) - \mu_1 R - R\theta_2\delta_B B\Big(\frac{T_{h_0}}{T_{h_2}}\Big) + R\mu_2,\\ &= (\theta_1\delta_m I_m - \theta_2\delta_B BR)\Big(\frac{T_{h_0}}{T_{h_2}}\Big) - R(\mu_1 - \mu_2). \qquad \text{(S.3)}\end{aligned}$$

Sensitivity analysis of the immune response parameters

Additional insights into the dynamics of the basic mathematical model can be obtained from the basic model disease reproduction number, R_0 (expression S.1), and carrying out sensitivity analysis. The value of R_0 determines whether infection will persist or is cleared . We find that the rate of infection of macrophages by bacteria (k_i), bursting rate of infected macrophages (k_b) and the

amount of bacteria released in the extracellular environment (N_o) are the main factors that define kinetics of disease progression during MAP infection. The parameter k_m which models the killing of bacteria by macrophages prevent infection progression. This result can easily be derived through sensitivity analysis of R_0 with respect to these parameters, $\frac{X_i}{R_0}\frac{\partial R_0}{\partial X_i}$, where X_i are the parameters in the R_0 expression [2]. A positive normalised derivative indicates that increasing the value of the corresponding parameters will increase disease progression, while a negative value implies suppression of infection progression.

Sensitivity analysis for infection (k_i, k_b, μ_B) and immune (k_m, k_i, θ_1, θ_2, δ_m, δ_B, μ_1, μ_2) parameters was carried out using the LHS method [3] at the time when the ratio of Th1/Th2 response reaches 1 as the output variable (Figure S1). Sensitivity analysis identified parameters that contribute the most to the timing of the Th1/Th2 switch including the decay rates of Th1 and Th2 cells (μ_1 and μ_2), rates at which Th0 cells differentiate into either Th1 or Th2 cells (δ_m and δ_B), clonal expansion factors (θ_1 and θ_2), and rate of bursting of infected macrophages, k_b.

Using the equation for R (S.3), sensitivity analysis was carried out to determine parameters that have significant influence to the Th1 to Th2 switch (Figure S1).

Sensitivity Analysis of R_0

Sensitivity indices for the basic model disease reproduction number, R_0, were evaluated. Sensitivity indices allows us to measure the relative change in R_0 with respect to its parameters. The normalised forward senstivity of a variable to a parameter is the ratio of the relative change in the variable to the relative change in the parameter [2]. The normalised forward sensitivity index of a variable, u, that depends differentiably on a parameter, p, is defined as

$$\mathcal{I}_p^u := \frac{\partial u}{\partial p} \times \frac{p}{u}.$$

Therefore, we can derive analytical expressions of the sensitivity indices of R_0 to be given by

$$\mathcal{I}_{X_i}^{R_0} = \frac{\partial R_0}{\partial X_i} \times \frac{X_i}{R_0},$$

where X_i are the eight parameters in the expression of R_0 and are evaluated to be

$$\begin{aligned}
\mathcal{I}_{No}^{R_0} &= 1, & \mathcal{I}_{k_b}^{R_0} &= \frac{\mu_i}{k_b + \mu_i}, \\
\mathcal{I}_{\mu_i}^{R_0} &= \frac{-\mu_i}{k_b + \mu_i}, & \mathcal{I}_{k_i}^{R_0} &= \frac{\sigma_m k_m + \mu_m \mu_B}{\sigma_m(k_i + k_m) + \mu_m \mu_B}, \\
\mathcal{I}_{\mu_B}^{R_0} &= \frac{-\mu_B \mu_m}{\sigma_m(k_i + k_m) + \mu_m \mu_B}, & \mathcal{I}_{k_m}^{R_0} &= \frac{-\sigma_m k_m}{\sigma_m(k_i + k_m) + \mu_m \mu_B}, \\
\mathcal{I}_{\mu_m}^{R_0} &= \frac{-\mu_m \mu_B}{\sigma_m(k_i + k_m) + \mu_m \mu_B}, & \mathcal{I}_{\sigma_m}^{R_0} &= \frac{\mu_B \mu_m}{\sigma_m(k_i + k_m) + \mu_m \mu_B}.
\end{aligned} \tag{S.4}$$

Parameter	Sensitivity index
N_o	1
k_b	0.96
μ_i	- 0.96
k_i	0.081
k_m	-0.058
σ_m	0.023
μ_B	-0.023
μ_m	-0.023

Table S1: **Sensitivity indices for** R_0. The parameters are ranked from the most sensitivity to the least. Increasing the value of a parameter with a positive index results in the increase of the R_0 value, hence infection progression, while increasing the numeric value of a parameter with a negative index will reduce infection progression. Parameters values used to calculate the sensitivity indices are given in Table 1.

N_o, k_b and k_i are the most sensitivity parameters that favour infection progression. Since $\mathcal{I}_{N_o}^{R_0} = 1$, therefore increasing (or decreasing) N_o by 10% will increase (or decrease) R_0 by 10%, while increasing k_b by 10% will increase R_0 by 9.6%.

Alternative models

The basic mathematical model of the MAP infection predicts that the classical Th1 to Th2 switch occurs late in infection when the rate of removal of extracellular bacteria is relatively slow, the burst size N_o is small ($N_o \approx 100$), and the decay rates of the Th1/Th2 responses are similar ($\mu_1 \approx \mu_2$). Under these circumstances there is a slow build-up of bacteria in the host. Here we investigate how additional immunological mechanisms may influence the timing of the Th1/Th2 switch if extracellular bacteria are short-lived. These mechanisms include inhibition of the Th1 cell differentiation by Th2 effectors, proliferation of effector T cells at the site of infection, and functional exhaustion of MAP-specific Th1 responses (Figure S2).

Differentiation cross inhibition and Th1/Th2 switch

There is strong evidence that cytokines produced by Th2 effectors skew differentiation of Th0 cells towards Th2 phenotype and suppress differentiation of cells into Th1 effectors (and *vice versa*) [4–7]. It is therefore possible that the switch from Th1 to Th2 response during MAP infection is due to suppression of the initially dominant Th1 response by Th2 effectors. To investigate this hypothesis we modified the terms for the generation of Th1 and Th2 subsets in Eqns. (4)-6) to $\dfrac{\delta_m I_m T_{h_0}}{1 + h_2 T_{h_2}}$ and $\dfrac{\delta_B B T_{h_0}}{1 + h_1 T_{h_1}}$, respectively, where h_1 and h_2 are inhibition constants. Interestingly, under conditions of a rapid removal of bacteria from extracellular environment when the classical switch is not observed (Figure 3B), inhibition of Th0 cell differentiation into Th1 subset by Th2 effectors allow for the loss of the protective Th1 response (Figure S3A). Increasing differentiation inhibition of Th1 response by Th2 effectors results in reduced Th1 cell population and increased growth of the Th2 subset of immune response. Reduced production of Th1 cells from differentiation will gradually weaken the protective immunity, which allows bacteria accumulation and disease progression. It should be noted, however, that if efficiency of suppression of Th2 cell differentiation by Th1 cells is high (h_1 is large) then the switch will not be observed even under the conditions of slow removal of extracellular

bacteria (results not shown). Thus, influence of Th1 and Th2 responses on differentiation of naïve CD4 T cells into effectors has a large impact on the kinetics of the Th1/Th2 switch.

Maintenance of committed effectors at the site of infection by proliferation

In our basic mathematical model we assumed that MAP-specific effector T cell responses are maintained at the site of infection by continuous recruitment of differentiated cells to the site of infection. However, it is possible that on site proliferation of effector T cells may contribute to the size of the Th1 and Th2 responses [4, 7–9]. To investigate whether effector T cell proliferation at the site of infection may influence the kinetics of the Th1/Th2 switch we extended the mathematical model. As in the case with differentiation of Th0 cells into Th1/Th2 effectors, we assume that proliferation of MAP-specific Th1 cells is mainly driven by the density of infected macrophages and that of Th2 cells by the density of extracellular bacteria. We thus add the following proliferation terms $\frac{\alpha_1 I_m T_{h_1}}{I_m + T_1}$ and $\frac{\alpha_2 B T_{h_2}}{B + T_2}$ to Eqns. (5) and (6) for Th1 and Th2 responses, respectively. Here α_1 and α_2 are the maximal rates of proliferation of Th1 and Th2 cells and T_1 and T_2 are half-saturation constants.

Proliferation of effector Th cells at the site of infection has a large impact on the kinetics of the switch, and sensitivity of the Th1 and Th2 subsets to the local antigen concentrations determined by the parameters T_1 and T_2, plays the major role. In the case of short-lived extracellular bacteria, if only minute amounts of free bacteria are sufficient to drive Th2 cell proliferation, the Th1/Th2 switch will occur (Figure S3B). On the other hand, if Th1 cell sensitivity for the antigen is low (low value of T_1), the Th1/Th2 switch may not occur even if extracellular bacteria are long-lived (results not shown). Thus, the specific details of how antigen availability influence the rate of proliferation of MAP-specific CD4 T cells are important in determining the likelihood and kinetics of the Th1/Th2 switch.

Exhaustion of the Th1 response

In chronic infections, T cells may become dysfunctional or exhausted [10–12]. Exhaustion has been mainly documented for virus-specific CD8 T cell responses and has been thought to arise when immune cells receive persistent stimulation. Exhausted T cells often fail to produce cytokines upon recognition of pathogen-infected cells. While the mechanistic details of how exhaustion develops especially for antigen-specific CD4 T cells are not fully understood, this mechanism may explain why Th1 responses are lost over the course of MAP infection. We investigated if exhaustion of the MAP-specific Th1 response may be responsible for the Th1/Th2 switch and disease progression. To model cell exhaustion, we assume an additional death term in Eqn. (5) for Th1 cells, $\nu T_{h_1}\mathcal{F}(I_m(t))$, where $\mathcal{F}(I_m(t)) = \int_0^t I_m(\tau)\mathrm{d}\tau$ [12,13]. The parameter, ν, is the rate of Th1 immune cells exhaustion and $\mathcal{F}(I_m)$ is the memory that is associated with accumulation of the number of times that a Th1 cell encounters an infected macrophage [13].

As expected, the possibility of exhaustion of MAP-specific Th1 response naturally leads to the loss of Th1 cells and, as the consequence, accumulation of ineffective Th2 response (Figure S3C). This occurs irrespectively of the death rate of extracellular bacteria suggesting that in this model the loss of protective Th1 response is the consequence of the disease progression in MAP-infected animals.

References

1. C. Castillo-Chavez, S. Blower, P. van den Driessche, D. Kirschner, and A.-A. Yakubu. *Mathematical approaches for emerging and reemerging infectious diseases.* Springer-Verlag, New York, 2002.

2. Nakul Chitnis, James M Hyman, and Jim M Cushing. Determining important parameters in the spread of malaria through the sensitivity analysis of a mathematical model. *Bulletin of mathematical biology*, 70(5):1272–1296, 2008.

3. Simeone Marino, Ian B Hogue, Christian J Ray, and Denise E Kirs chner. A methodology for performing global uncertainty and sensitivity analysis in systems biology. *J Theor Biol*, 254(1):178–196, 2008.

4. A. OŠGarra. T-cell differentiation: Commitment factors for T helper cells. *Current Biology*, 10(13):R492–R494, 2000.

5. E. Murphy, K. Shibuya, N. Hosken, P. Openshaw, V. Maino, K. Davis, K. Murphy, and A. O'Garra. Reversibility of T helper 1 and 2 populations is lost after long-term stimulation. *The Journal of experimental medicine*, 183(3):901, 1996.

6. S.J. Szabo, N.G. Jacobson, A.S. Dighe, U. Gubler, and K.M. Murphy. Developmental commitment to the Th2 lineage by extinction of IL-12 signaling. *Immunity*, 2(6):665–675, 1995.

7. H. Chakir, H. Wang, D.E. Lefebvre, J. Webb, and F.W. Scott. T-bet/GATA-3 ratio as a measure of the Th1/Th2 cytokine profile in mixed cell populations: predominant role of GATA-3. *Journal of immunological methods*, 278(1):157–169, 2003.

8. J. Zhu, H. Yamane, J. Cote-Sierra, L. Guo, and W.E. Paul. Gata-3 promotes Th2 responses through three different mechanisms: induction of Th2 cytokine production, selective growth of Th2 cells and inhibition of Th1 cell-specific factors. *Cell research*, 16(1):3–10, 2006.

9. C. Doebis, A. Menning, K. Neumann, S. Ghani, K. Schlawe, U. Lauer, A. Hamann, J. Huehn, and U. Syrbe. Accumulation and local proliferation of antigen-specific CD4&; T cells in antigen-bearing tissue. *Immunology and Cell Biology*, 89(4):566–572, 2010.

10. A. Koets, V. Rutten, A. Hoek, F. Van Mil, K. Müller, D. Bakker, E. Gruys, and W. Van Eden. Progressive bovine paratuberculosis is associated with local loss of CD4+ T cells, increased frequency of $\gamma\delta$ T cells, and related changes in T-cell function. *Infection and immunity*, 70(7):3856–3864, 2002.

11. E.J. Wherry, J.N. Blattman, K. Murali-Krishna, R. Van Der Most, and R. Ahmed. Viral persistence alters CD8 T-cell immunodominance and tissue distribution and results in distinct stages of functional impairment. *Journal of virology*, 77(8):4911–4927, 2003.

12. P.L.F. Johnson, B.F. Kochin, M.S. McAfee, I.M. Stromnes, R.R. Regoes, R. Ahmed, J.N. Blattman, and R. Antia. Vaccination alters the balance between protective immunity, exhaustion, escape, and death in chronic infections. *Journal of virology*, 85(11):5565–5570, 2011.

13. GA Bocharov. Modelling the dynamics of LCMV infection in mice: conventional and exhaustive CTL responses. *Journal of theoretical biology*, 192(3):283–308, 1998.
